# Concomitant Diagnosis of Acute Promyelocytic Leukemia and Type 2 Diabetes Mellitus in a Child: Report of a Rare Case

**DOI:** 10.7759/cureus.111647

**Published:** 2026-06-28

**Authors:** Ikram El Hachmi, Ayad Ghanam, Aziza Elouali, Rachid Seddik, Maria Rkain

**Affiliations:** 1 Department of Pediatrics, Mohammed VI University Hospital, Mohammed First University, Oujda, MAR; 2 Laboratory of Biological Hematology, Mohammed First University, Oujda, MAR

**Keywords:** acute myeloid leukemia, acute promyelocytic leukemia, childhood diabetes, hyperglycemia, pediatric oncology, pml-rara, type 2 diabetes mellitus

## Abstract

Acute promyelocytic leukemia (APL) is a distinct subtype of acute myeloid leukemia characterized by the PML-RARA fusion gene and a high risk of coagulopathy. Type 2 diabetes mellitus (T2DM) is increasingly diagnosed in children and adolescents. To our knowledge, the concomitant diagnosis of these two conditions in the pediatric population has not been previously reported.

We report the case of a 10-year-old girl presenting with fatigue, abdominal pain, and fever. Laboratory investigations revealed severe thrombocytopenia, leukocytosis, and 63% circulating blasts on peripheral blood smear. Bone marrow aspiration, immunophenotyping, and molecular analysis confirmed APL through detection of the t(15;17)(q24.1;q21.2) translocation and PML-RARA fusion gene. Based on a white blood cell count of 16.9 × 10⁹/L and a platelet count of 10 × 10⁹/L, the patient was classified as high-risk APL according to the Sanz classification. Severe disseminated intravascular coagulation was present at diagnosis. Concurrent metabolic evaluation demonstrated marked hyperglycemia, elevated HbA1c (8.1%), hyperinsulinemia, atherogenic dyslipidemia, and negative pancreatic autoantibodies, supporting a concomitant diagnosis of T2DM in the setting of overweight status, acanthosis nigricans, and a positive family history. Treatment was initiated according to the AML-MA 2011 induction protocol, comprising cytarabine and daunorubicin combined with all-trans retinoic acid (ATRA), intensive transfusion support, and a basal-bolus insulin regimen. At 18 months of follow-up, the patient remained in complete hematological remission under joint hematological and endocrinological surveillance.

This case highlights the importance of considering metabolic disorders in children with hematologic malignancies who present with risk factors for insulin resistance. Emerging evidence suggests potential biological links between metabolic dysregulation and hematological malignancies. Early multidisciplinary management enabled successful control of both conditions and a favorable clinical outcome.

## Introduction

Acute promyelocytic leukemia (APL) is a distinct subtype of acute myeloid leukemia (AML), accounting for approximately 15% of AML cases [1,2]. It is characterized by the t(15;17)(q24.1;q21.2) translocation and the resulting PML-RARA fusion gene. Although associated with a high risk of life-threatening coagulopathy and hemorrhagic complications, APL has become one of the most curable forms of acute leukemia since the introduction of all-trans retinoic acid (ATRA) and arsenic trioxide (ATO) [2].

Type 2 diabetes mellitus (T2DM) is increasingly recognized in children and adolescents worldwide, with adolescence representing a particularly vulnerable period because of the physiological insulin resistance associated with puberty [3]. The growing burden of youth-onset T2DM has led to the development of dedicated diagnostic and management recommendations by both the International Society for Pediatric and Adolescent Diabetes (ISPAD) and the American Diabetes Association (ADA) [4,5].

We report an unusual case of simultaneous diagnosis of APL and T2DM in a 10-year-old girl. While both conditions are increasingly encountered in pediatric practice, the relationship between diabetes and leukemia in children has been explored primarily in two contexts: treatment-related hyperglycemia occurring during chemotherapy [6] and the potential association between type 2 diabetes mellitus and the risk of acute myeloid leukemia, mainly reported in adult populations [7,8]. However, we were unable to identify any previous report describing the simultaneous diagnosis of APL and T2DM in a child. The de novo coexistence of these two conditions at presentation, as observed in our patient, therefore appears to represent a previously undescribed clinical scenario. 

## Case presentation

A 10-year-old girl, born to non-consanguineous parents, was admitted to our pediatric department for abdominal pain, progressive asthenia, and fever. Her family history was notable for maternal type 2 diabetes mellitus. No family history of obesity, hypertension, dyslipidemia, metabolic syndrome, or hematological malignancies was reported. 

On admission, physical examination revealed marked cutaneous and mucosal pallor, multiple ecchymotic lesions, and acanthosis nigricans involving the neck. Anthropometric assessment showed a weight of 46 kg, a height of 148 cm, and a body mass index (BMI) of 21 kg/m², corresponding to the 85th-95th percentile for age and sex according to CDC growth charts (Z-score +1.37), consistent with overweight status as defined by both CDC and WHO criteria [9,10]. The patient was at Tanner stage S2P2, consistent with normal pubertal development for her age. 

**Figure 1:**
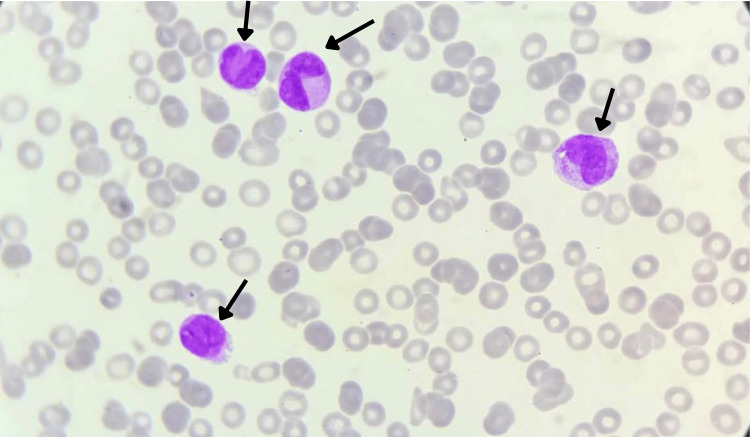
Peripheral blood smear at diagnosis showing numerous circulating abnormal promyelocytes/blasts (arrows), raising suspicion for acute myeloid leukemia

Initial laboratory investigations demonstrated anemia (hemoglobin 8.9 g/dL), severe thrombocytopenia (10 × 10⁹/L), and leukocytosis (16.9 × 10⁹/L). Peripheral blood smear revealed 63% circulating blasts (Figure 1).

**Figure 2:**
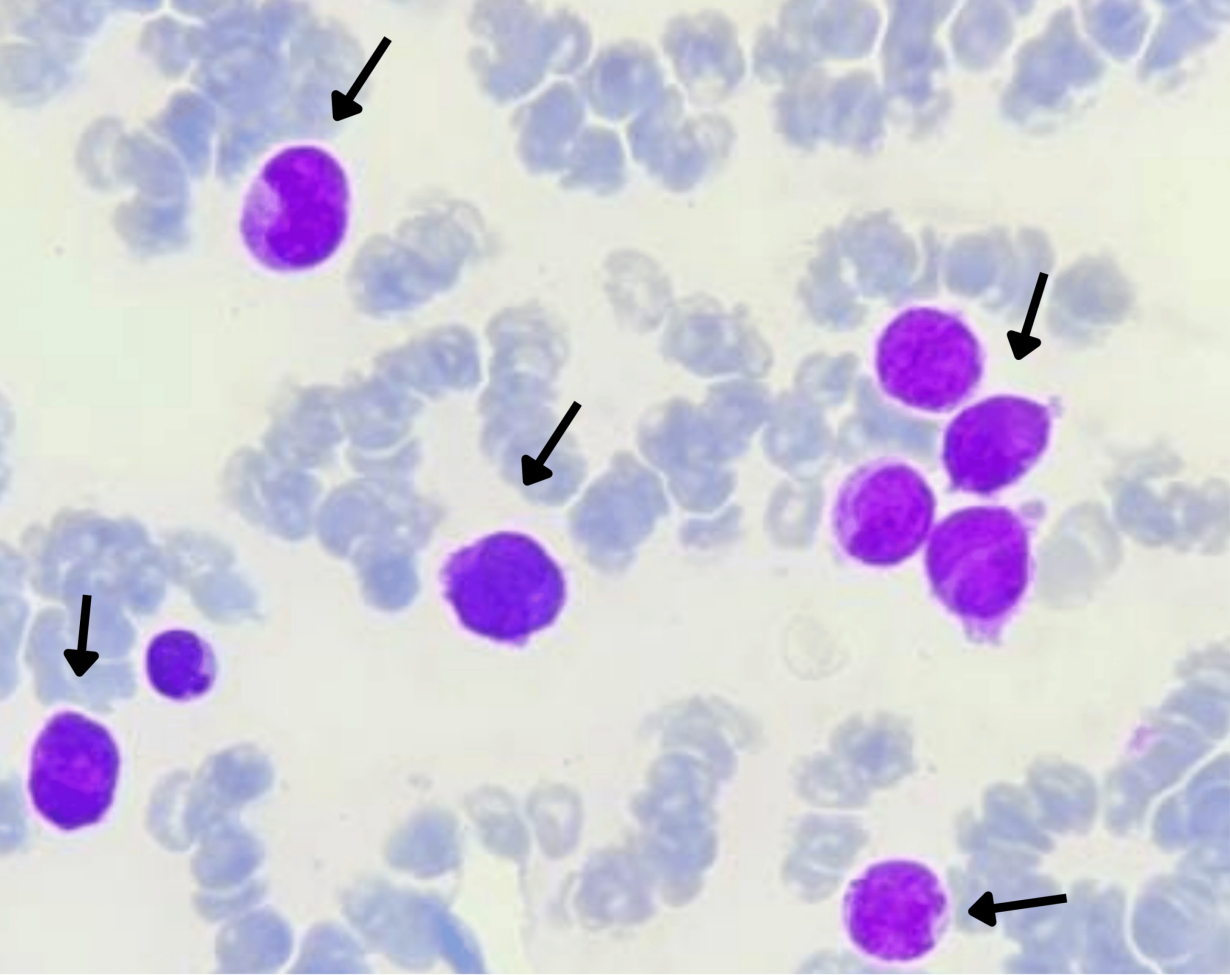
Bone marrow aspirate demonstrating infiltration by abnormal promyelocytes (arrows) Abnormal promyelocytes (arrows) characterized by large cells with irregular nuclei and abundant cytoplasm, findings consistent with acute promyelocytic leukemia (AML-M3 subtype). The diagnosis was confirmed by immunophenotyping and detection of the PML-RARA rearrangement.

Bone marrow aspiration demonstrated infiltration by abnormal promyelocytes (Figure 2). Flow cytometric immunophenotyping demonstrated positivity for CD33, CD13, weak CD117 expression, and intracellular myeloperoxidase, with negativity for CD34 and HLA-DR, supporting the diagnosis of APL. Molecular analysis confirmed the presence of the t(15;17)(q24.1;q21.2) translocation resulting in the PML-RARA fusion gene, establishing the diagnosis of acute promyelocytic leukemia (APL, AML-M3 subtype). Based on a white blood cell count of 16.9 × 10⁹/L and a platelet count of 10 × 10⁹/L, the patient was classified as high-risk APL according to the Sanz risk classification [11].

At diagnosis, the patient also exhibited disseminated intravascular coagulation, with severe thrombocytopenia, hypofibrinogenemia (1.2 g/L), a prothrombin time of 54%, a prolonged activated partial thromboplastin time of 39 seconds (ratio 1.56), an INR of 1.02, and markedly elevated D-dimer levels (12 mg/L). Lactate dehydrogenase was elevated at 1207 U/L. Cerebrospinal fluid examination showed no evidence of leukemic involvement. Abdominal ultrasonography revealed splenomegaly, whereas baseline echocardiography demonstrated preserved cardiac function with a left ventricular ejection fraction of 62%.

Concomitantly, metabolic evaluation performed during the initial diagnostic workup revealed marked hyperglycemia, with capillary blood glucose levels ranging from 300 to 500 mg/dL (16.7-27.8 mmol/L) and glycosuria without ketonuria and a serum bicarbonate level of 21 mmol/L, findings not consistent with diabetic ketoacidosis. Glycated hemoglobin was elevated at 8.1%, indicating chronic dysglycemia. Further investigations showed a C-peptide level of 2.65 ng/mL and elevated insulin levels (28.2 μIU/mL). Lipid profile demonstrated hypertriglyceridemia (3.35 g/L), elevated total cholesterol (2.26 g/L), low high-density lipoprotein (HDL) cholesterol (0.28 g/L), and low-density lipoprotein (LDL) cholesterol of 1.31 g/L. Autoimmune testing was negative for anti-glutamic acid decarboxylase (anti-GAD), anti-islet antigen-2 (anti-IA2), anti-islet cell (ICA), and anti-zinc transporter 8 (ZnT8) antibodies. The patient's laboratory findings at diagnosis are summarized in Table 1. 

**Table 1:** Laboratory findings at diagnosis HbA1c - glycated hemoglobin; HDL - high-density lipoprotein; LDL - low-density lipoprotein

Parameter (unit)	Result	Reference range
Hemoglobin (g/dL)	8.9	11.5-15.5
White blood cell count (×10⁹/L)	16.9	4.5-13.5
Platelet count (×10⁹/L)	10	150-450
Peripheral blasts (%)	63	0
Fibrinogen (g/L)	1.2	2.0-4.0
D-dimers (mg/L)	12	<0.5
Lactate dehydrogenase (U/L)	1207	120-300
Blood glucose (g/L)	3–5	0.70-1.10
HbA1c (%)	8.1	<5.7
Insulin (μIU/mL)	28.2	4-20
C-peptide (ng/mL)	2.65	0.78-5.19
Total cholesterol (g/L)	2.26	<2.00
Triglycerides (g/L)	3.35	<1.50
HDL cholesterol (g/L)	0.28	>0.40
LDL cholesterol (g/L)	1.31	<1.30

Based on the patient's overweight status, acanthosis nigricans, positive family history of type 2 diabetes mellitus, elevated insulin levels, a C-peptide level within the reference range, dyslipidemia, and negative pancreatic autoantibodies, a diagnosis of type 2 diabetes mellitus was established concomitantly with acute promyelocytic leukemia.

Treatment was initiated according to the national AML-MA2011 protocol, supplemented with all-trans retinoic acid (ATRA). Induction therapy consisted of two courses combining ATRA with cytarabine- and daunorubicin-based chemotherapy; etoposide was added during the second induction course. Consolidation therapy was based on high-dose cytarabine and daunorubicin. Maintenance therapy consisted of intermittent ATRA administered for 15 days every three months. Central nervous system (CNS) prophylaxis was provided through intrathecal chemotherapy throughout treatment. Supportive care included repeated transfusions of platelet concentrates, fresh frozen plasma, and packed red blood cells. 

Glycemic control was achieved using a basal-bolus insulin regimen consisting of insulin glargine administered once daily and insulin glulisine administered before meals. Capillary blood glucose was monitored before meals and at bedtime. Target blood glucose levels were maintained between 100 and 180 mg/dL (5.6-10.0 mmol/L), consistent with general inpatient glycemic management targets recommended by the American Diabetes Association for noncritically ill individuals [12]. Insulin doses were adjusted according to serial glucose measurements throughout treatment. Insulin requirements ranged from approximately 0.7 to 1 U/kg/day, with progressive improvement in glycemic control. 

At the most recent follow-up, approximately 18 months after diagnosis, the patient remained in complete hematological remission and continued regular endocrinological follow-up. Type 2 diabetes mellitus remained managed with a basal-bolus insulin regimen. Metabolic reassessment showed significant improvement in glycemic control, with HbA1c at 6.2%. Anthropometric assessment showed a weight of 54 kg and a height of 156 cm (BMI 22.2 kg/m²; BMI-for-age Z-score +1.11), consistent with persistent overweight status according to both CDC and WHO criteria. Nevertheless, the BMI-for-age Z-score improved compared with baseline (+1.37 at diagnosis). Complete blood count and biochemical parameters had normalized.

## Discussion

Acute promyelocytic leukemia (APL) is a rare subtype of acute myeloid leukemia affecting both adult and pediatric populations, accounting for about 15% of AML cases [1,2]. APL is characterized by the translocation t(15;17)(q24.1;q21.2), resulting in the PML-RARA fusion gene [1]. Unlike other AML subtypes, APL is associated with a high risk of coagulopathy and life-threatening hemorrhagic complications [2]. Since the introduction of all-trans retinoic acid (ATRA) in the 1980s and arsenic trioxide (ATO) in the 1990s, patient outcomes have improved dramatically, making APL the most curable subtype of acute leukemia, with long-term disease-free survival approaching 90% [1]. 

Disseminated intravascular coagulation (DIC) is the most feared complication of APL and remains the leading cause of early mortality [2]. In our patient, severe DIC manifested as profound thrombocytopenia, hypofibrinogenemia, and markedly elevated D-dimer levels. Management included all-trans retinoic acid (ATRA) combined with intensive transfusion support, including platelet concentrates, fresh frozen plasma, and packed red blood cells. 

Type 2 diabetes mellitus (T2DM), once considered a disease of adulthood, is increasingly diagnosed in children and adolescents worldwide. Over the past three decades, the prevalence of youth-onset T2DM has increased two- to threefold, largely driven by rising rates of obesity, sedentary lifestyles, and unhealthy dietary habits. Adolescence represents a particularly vulnerable period because physiological insulin resistance associated with puberty may accelerate the progression from prediabetes to overt diabetes in predisposed individuals [3]. The diagnosis of T2DM in our patient was supported by several converging findings, including overweight status with acanthosis nigricans, a clinical marker of insulin resistance, a first-degree family history of T2DM, a C-peptide level within the reference range, and negative pancreatic autoantibodies (anti-GAD, anti-IA2, ICA, and ZnT8). Collectively, these findings strongly supported the diagnosis of T2DM and argued against autoimmune diabetes. This profile is consistent with the diagnostic criteria outlined in the ISPAD 2024 Clinical Practice Consensus Guidelines [4] and the 2024 ADA Standards of Care [5]. 

Although the coexistence of APL and T2DM in our patient may be coincidental, emerging evidence suggests potential biological links between hematologic malignancies and metabolic disorders. Obesity and chronic hyperglycemia are known to promote a pro-inflammatory and pro-proliferative environment through mechanisms involving hyperinsulinemia, insulin-like growth factor-1 (IGF-1), and metabolic dysregulation [13]. 

In parallel, recent genomic and pathway analyses have identified shared susceptibility genes and dysregulated pathways involving insulin signaling, AMPK, mTOR, and PI3K, highlighting the role of metabolic reprogramming in both AML and T2DM [7]. Supporting this hypothesis, a meta-analysis of 18 studies involving 10,516 leukemia cases among more than 4 million individuals with diabetes found that leukemia risk was increased in patients with T2DM but not in those with type 1 diabetes [8]. In addition, AML has been reported to occur more frequently in individuals with T2DM, with a standardized incidence ratio of 1.36 (95% CI, 1.26-1.47) compared with the general population [7]. These findings raise the possibility that common metabolic disturbances may contribute to the coexistence of hematologic malignancies and type 2 diabetes, although the underlying mechanisms remain incompletely understood. 

Previous pediatric literature has primarily described treatment-related transient hyperglycemia or secondary diabetes occurring during chemotherapy for acute leukemia, particularly in association with corticosteroids and asparaginase exposure [6]. Co-occurrence with type 1 diabetes mellitus (T1DM) has also been reported, often in the context of asparaginase-induced pancreatitis or as sequential rather than simultaneous diagnoses [14,15]. In contrast, our patient presented with a de novo concomitant diagnosis of high-risk APL and T2DM at presentation, prior to initiation of antileukemic therapy. While several epidemiological studies in adults have identified T2DM as a potential risk factor for AML [8], we were unable to identify any previous report describing the simultaneous diagnosis of APL and T2DM in a child. To our knowledge, the presentation observed in our patient has not been previously reported in the pediatric literature. 

Beyond its potential role in cancer susceptibility, diabetes may also influence the clinical course of malignant diseases through metabolic and treatment-related mechanisms [16]. The optimal management of T2DM in patients with newly diagnosed leukemia has not been clearly established. Treatment decisions should consider the severity of hyperglycemia, the patient's clinical condition, and potential interactions with anticancer therapies [17]. In our patient, a basal-bolus insulin regimen was preferred because it allowed rapid and flexible glycemic control during the acute phase of leukemia treatment. This approach is consistent with current recommendations advocating individualized antihyperglycemic management in patients undergoing cancer therapy [17].

At 18 months of follow-up, the patient remains in complete hematological remission with normalized biological parameters under joint hematological and endocrinological surveillance. This favorable outcome highlights the feasibility of successfully managing both conditions simultaneously through a multidisciplinary approach. Nonetheless, long-term vigilance remains essential. A recent meta-analysis estimated the pooled prevalence of metabolic syndrome among pediatric leukemia survivors at 13%, underscoring the importance of continued metabolic monitoring in patients with pre-existing risk factors [18]. Given her history of T2DM, overweight status, and dyslipidemia, regular assessment of glycemic control, lipid profile, and diabetes-related complications should remain an integral component of her long-term follow-up.

## Conclusions

The simultaneous diagnosis of acute promyelocytic leukemia and type 2 diabetes mellitus in a pediatric patient appears to be exceptionally rare and, to our knowledge, has not been previously reported in the pediatric literature. This unusual association poses unique diagnostic and therapeutic challenges. The present case highlights the importance of comprehensive metabolic evaluation in children presenting with hematologic malignancies, particularly in the presence of overweight status, a family history of T2DM, and clinical features of insulin resistance.

Although the coexistence of these two conditions may be coincidental, emerging evidence suggests potential biological interactions between metabolic dysregulation and leukemogenesis. Despite the complexity of this dual diagnosis, a coordinated multidisciplinary approach enabled successful management of both conditions, with complete hematological remission achieved at 18 months of follow-up. Long-term endocrinological surveillance remains essential to monitor glycemic control, metabolic complications, and cardiovascular risk factors throughout survivorship care. 
